# Idiopathic Orbital Inflammation Syndrome with Retro-Orbital Involvement: A Retrospective Study of Eight Patients

**DOI:** 10.1371/journal.pone.0057126

**Published:** 2013-02-21

**Authors:** Yumei Li, Gerald Lip, Vincent Chong, Jianhua Yuan, Zhongxiang Ding

**Affiliations:** 1 Department of Radiology, Zhejiang Provincial People's Hospital, Hangzhou, Zhejiang, P. R. China; 2 Department of Diagnostic Imaging, National University Health System, Yong Loo Lin School of Medicine, National University of Singapore, Singapore, Singapore; Oregon Health & Science University, United States of America

## Abstract

**Background:**

The aim of this retrospective study was to document the clinical findings and radiological features of idiopathic orbital inflammation syndrome with retro-orbital involvement.

**Methods:**

We searched for ophthalmological patients who received orbital imaging at Zhejiang Provincial People's Hospital between October 2003 and April 2010. Seventy-three patients were diagnosed with idiopathic orbital inflammation syndrome based on clinicoradiological features, with pathological confirmation of nonspecific inflammatory conditions in 47 patients. Eight patients (11%) had MRI or CT evidence of retro-orbital involvement. All 8 patients were diagnosed with idiopathic orbital inflammation syndrome after biopsy of the orbital lesion. MR images were obtained for all 8 patients; 3 patients also had a contrast-enhanced CT scan.

**Results:**

Seven out of 8 patients with retro-orbital involvement also had orbital apex lesions. Of the 65 patients without retro-orbital involvement, 19 had orbital apex lesions. The difference in the number of patients with orbital apex lesions between the two populations was significant (Fisher exact test *P* = .002). In all 8 patients with retro-orbital involvement, the inflammation spread through the superior orbital fissure. The retro-orbital lesions were isointense to grey matter on T1-weighted images, hypointense on T2-weighted images, and displayed uniform contrast enhancement; on contrast-enhanced CT scans, they were hyperdense relative to the contralateral mirror area and had radiological contours that were similar to those seen on MR images. The diffuse inflammation with marked sclerosis and hyalinization that we observed in the patients with retro-orbital involvement is consistent with the diagnosis of the sclerosing subtype of idiopathic orbital inflammation syndrome. All 8 patients also complained of mild to moderate periorbital pain (headache).

**Conclusions:**

In patients with idiopathic orbital inflammation syndrome, it is important to perform MRI and CT scans to identify possible retro-orbital involvement. Retro-orbital involvement is more frequent when the lesion is present in the orbital apex.

## Introduction

Idiopathic orbital inflammation syndrome (IOIS), also known as idiopathic orbital pseudotumor (first described by Birch-Hirschfield in 1905), is a benign, noninfective, inflammatory condition of the orbit with no identifiable local or systemic causes [1]. It is the third most common orbital disease after thyroid orbitopathy and lymphoproliferative disorder [2]. Lesions are commonly restricted to the orbit. However, nonspecific inflammatory tissue of the orbit may extend into the adjacent retro-orbital structures through one or more foramina, including the superior orbital fissure, optic canal, and inferior orbital fissure. Herein, we report the clinical presentation, radiological features, and pathological findings of 8 patients with IOIS with retro-orbital involvement.

## Methods

### MRI and CT Criteria for the Diagnosis of Retro-orbital Involvement

Two experienced head and neck radiologists evaluated the MRI and CT images for features of orbital and retro-orbital involvement. Retro-orbital involvement was diagnosed if any of the following radiological features were identified: 1) the presence of abnormal soft tissue that extends through the superior orbital fissure into the middle cranial fossa and obliterates the normal fatty plane; 2) an expansion of the ipsilateral cavernous sinus; and 3) a distant abnormal thickening or enhancement of the meninges, in continuity with the orbital lesion [3].

### Patients

We conducted a retrospective search for ophthalmological patients who had received orbital imaging at Zhejiang Provincial People's Hospital between October 2003 and April 2010. Seventy-three patients were diagnosed with IOIS. Of these 73 patients, the diagnosis in 26 (35.6%) was based on clinicoradiological features with no identifiable local or systemic causes. In the remaining 47 patients (64.4%), a biopsy of the orbital lesion excluded local or systemic disorders and allowed pathological confirmation of nonspecific inflammatory conditions. Sixty-five of 73 patients (89%) with IOIS had no evidence of retro-orbital involvement according to MR and CT imaging criteria. Nineteen of the 65 patients (29.2%) showed orbital apex involvement. Furthermore, we found cranial nerve palsies in 12 of these 19 patients (63.2%).

Eight of 73 patients (11%) had MRI or CT evidence of retro-orbital involvement. These 8 patients were all diagnosed with IOIS following biopsy of the orbital lesion that was associated with abnormal retro-orbital soft tissue. Of the 8 patients who had MRI or CT evidence of retro-orbital involvement, 5 were men and 3 were women, with a median age of 51.6 years (range, 34–72 years). All 8 patients had an insidious onset. The average duration of symptoms before presentation was 16 months (range, 5 weeks - 9 years). We recorded the clinical features and presentation including relative proptosis, decreased visual acuity, a relative afferent papillary defect, and cranial nerve palsies. Patients with IOIS with retro-orbital involvement underwent standard treatment with a high-dose oral steroid (systemic prednisone, 1.0–1.6 mg/kg of body weight per day) for 5 to 10 days followed by a tapering period for 6 to 8 weeks. Radiation treatment, surgical debulking, and other related therapies were refused by the patients if they did not respond to or were intolerant of systemic steroid therapy. The mean follow-up period was 19 months (range, 13–38 months). The study was approved by the ethics committee of Zhejiang Provincial People's Hospital and written consent was obtained from all patients.

### MRI and CT Examinations

MR images were obtained for all 8 patients; 3 patients also had a contrast-enhanced CT scan. MRI was performed on a 1.5 T (General Electric, Waukesha WI, USA) or a 3T superconducting system (Siemens Medical Solutions, Erlangen, Germany). The scanning range was from the upper margin of the corpus callosum to the lower edge of the maxillary sinus. Axial, coronal, and sagittal spin-echo T1W images, as well as axial T2W images were obtained. The imaging parameters were as follows: T1WI, TR = 300–500 ms and TE = 15–30 ms; T2WI, TR = 3000–4000 ms and TE = 100–120 ms; slice thickness = 5 mm. In addition, contrast-enhanced axial, coronal, and sagittal T1WI scans were performed with fat suppression; the patient position and imaging parameters were the same as for the T1WI scans without fat suppression. Gadopentetate dimeglumine (Gd-DTPA) was used as a contrast media (0.1 mmol/kg body weight). A contrast-enhanced orbital CT scan was obtained in 3 patients using a multidetector-row CT (MDCT) scanner (4-slice or 64-slice CT, Siemens Medical Solutions, Forchheim, Germany); CT scans were not performed without contrast. The following CT scan protocol was applied: slice collimation, 3 mm; tube voltage, 140 kV; tube current, 120 mAs. A total of 50 ml of iodinated contrast material (Omnipaque 350; Nycomed, Princeton NJ, USA) was administered using a power injector at a rate of 1.5–2.0 ml/sec, with a delay of 30 seconds. Axial images were acquired and reformatted to obtain 2-mm – to 3-mm-long coronal images along an axis perpendicular to the hard palate.

### Statistical Analysis

All statistical analyses were performed using SPSS software, Version 16.0 (SPSS, Chicago, IL, USA). Differences in the involvement of the orbital apex, the incidence of cranial nerve palsies between patients with and without MRI and CT scans, and evidence of retro-orbital involvement were compared using the Fisher exact test. *P* value<.05 were considered significant.

## Results

### Clinical Findings

At presentation, the most common symptom in the 8 patients was periorbital pain and headache (a moderate symptom in 3 patients and mild in 5). No patients in this study experienced severe pain. The next two most-frequent symptoms were cranial nerve palsies and decreased visual acuity, which occurred in 6 patients. Five patients had a relative proptosis of 2 mm or more, with a range of 2 mm to 5 mm (mean, 3.4 mm). Three patients had an abnormal optic disc: the discs of 2 patients were swollen, but in 1 patient, the disc was atrophic and flat. No concurrent autoimmune disease was diagnosed in any patient. Table 1 summarizes the clinical findings of the study population.

**Table 1:** The clinical characteristics of 8 patients with IOIS with retro-orbital involvement.

Patient No.	Age, y/gender	Proptosis measurement (mm)			Periorbital pain/headache	Visual acuity		Cranial nerve palsies	RAPD
		R	L	Relative proptosis		R	L		
1	34/M	13	16	3	+	20/10	20/35	−	−
2	52/M	14	19	5	+	20/30	20/190	L 3, 4, 5 and 6	+
3	46/W	12	12	0	+	20/20	20/15	R 3, 4 and 6	−
4	67/M	15	18	3	+	20/20	20/20	−	−
5	72/M	13	14	1	+	20/40	20/125	L 3, 4 and 6	+
6	41/W	14	16	2	+	20/20	20/80	L 3, 4 and 6	+
7	39/W	12	13	1	+	20/35	20/200	L 3, 4 and 6	+
8	62/M	18	14	4	+	20/60	20/20	R 3, 4 and 6	+

Abbreviations: CECT, contrast-enhanced CT; +, Positive; −, Negative; L, left; R, right; RAPD, relative afferent papillary defect; mm, millimeters; M, men; W, women; L 3, 4, 5 and 6, left oculomotor nerve, trochlear nerve, trigeminal nerve and abducens nerve.

### Imaging Findings

All 8 patients had a unilateral orbital lesion, located on the right in 2 patients and on the left in 6. Seven patients had orbital apex lesions; 4 had diffuse infiltration with orbital apex involvement, and in 3 a localized mass was detected in the orbital apex. The remaining patient had optic perineural infiltration (perineuritis with involvement of the outer dural sheath of the optic nerve and adjacent fat). Involvement of the orbital apex was more common in patients with retro-orbital involvement (7 of 8 patients) than in patients with no MRI or CT evidence of retro-orbital involvement (19 of 65 patients). Between these two populations, the differences in the frequency of orbital apex involvement was statistically significant (Fisher exact test *P* = .002). Despite the fact that the frequency of cranial nerve palsies in patients with IOIS with retro-orbital involvement (75%, 6 of 8 patients) was higher than in patients with IOIS with orbital apex involvement but no MRI or CT evidence of retro-orbital involvement (63%, 12 of 19 patients), the difference between the two populations was not significant (Fisher exact test *P* = .450).

**Figure 1:**
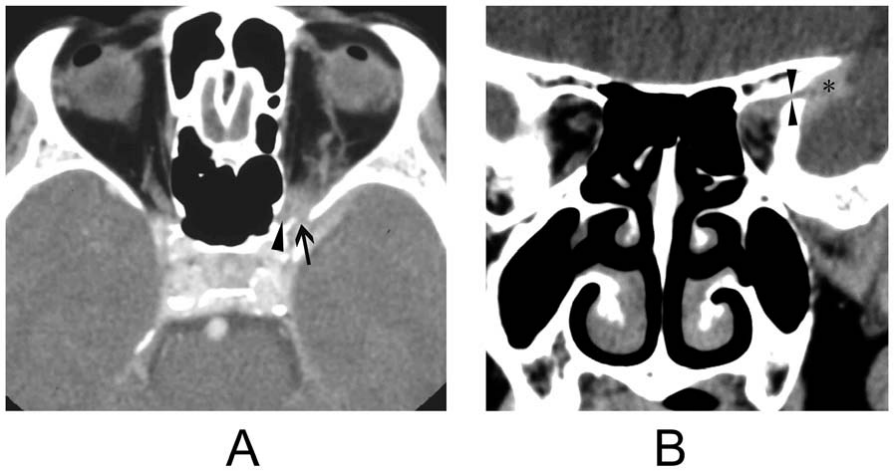
Idiopathic orbital inflammation syndrome with middle cranial fossa involvement. Figure 1A: Axial contrast-enhanced CT imaging reveals a lesion in the left orbital apex that extends through the superior orbital fissure (arrow) into ipsilateral middle cranial fossa. Note the involvement of the left optic canal (arrowhead). Figure 1B: Coronal contrast-enhanced CT imaging reveals a lesion in left orbital apex that extends through the superior orbital fissure (opposing arrowheads) into the ipsilateral middle cranial fossa (asterisk).

**Figure 2:**
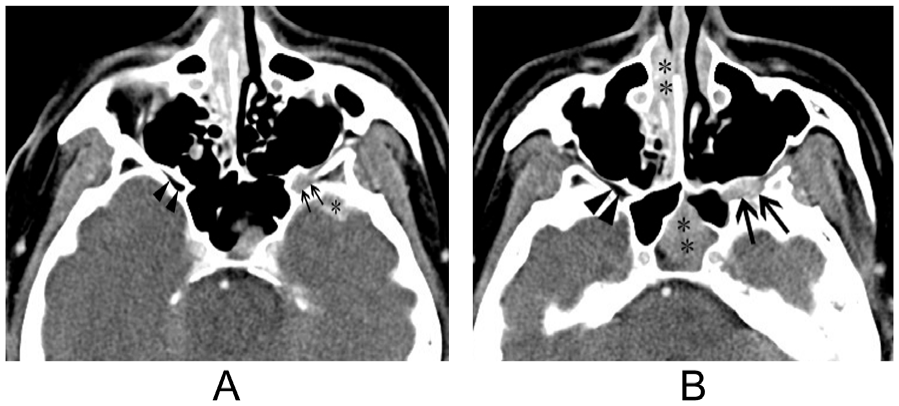
Idiopathic orbital inflammation syndrome with pterygopalatine fossa and middle cranial fossa involvement. Figure 2A: Axial contrast-enhanced CT imaging reveals enlargement of and abnormal soft tissue within the left inferior orbital fissure (arrows). Compare this to the normal inferior orbital fissure on the contralateral side (arrowheads). Note the involvement of the left middle cranial fossa (asterisk). Figure 2B: Axial contrast-enhanced CT imaging reveals enlargement of and abnormal soft tissue within the left pterygopalatine fossa (arrows). Compare this to the normal pterygopalatine fossa on the contralateral side (arrowheads). Note the sphenoidal and right ethmoidal sinusitis (asterisks).

**Figure 3:**
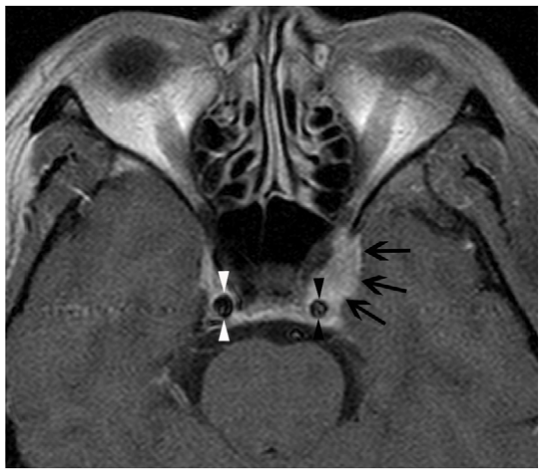
Idiopathic orbital inflammation syndrome with encasement and narrowing of the cavernous carotid artery. Axial fat-saturated contrast-enhanced T1w imaging reveals a lesion that extends through the ipsilateral superior orbital fissure into the left cavernous sinus (arrows), with encasement and narrowing of the cavernous carotid artery (black opposing arrowheads). Compare this to the normal carotid artery on the contralateral side (white opposing arrowheads).

Retro-orbital involvement of IOIS through the superior orbital fissure was found in all 8 patients (Figure 1). The lesion extended through the optic canal in 2 patients (Figure 1A) and through the inferior orbital fissure in 2 patients (Figure 2), The orbital lesion extended into the cavernous sinus in 6 patients, 2 of whom showed narrowing and encasement of the cavernous carotid artery (Figure 3). Retro-orbital involvement was also noted in the middle cranial fossa of 7 patients, the pterygopalatine fossa of 2 patients, and the infratemporal fossa of 1 patient.

**Figure 4:**
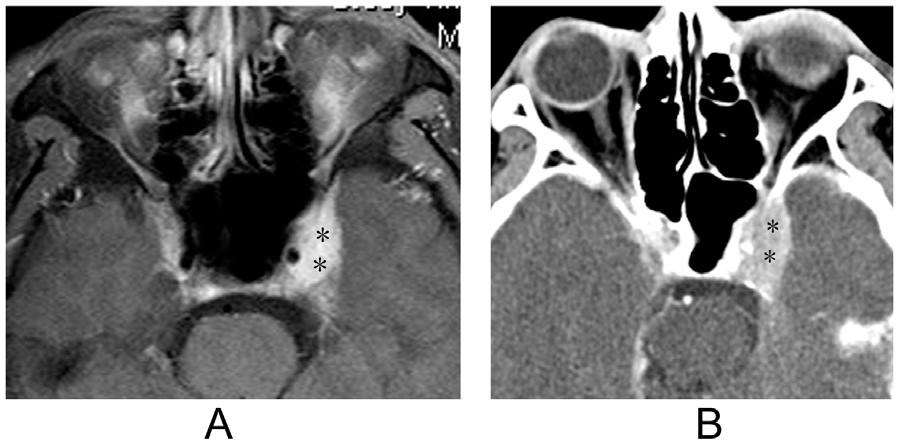
Idiopathic orbital inflammation syndrome with cavernous sinus involvement. Figure 4A: Axial fat-saturated contrast-enhanced T1w imaging reveals a lesion that extends into the left cavernous sinus (asterisks). Figure 4B: Axial contrast-enhanced CT imaging reveals enlargement of the left cavernous sinus. The radiological contours of the enlargement are similar to those observed with MR imaging (asterisks).

The retro-orbital lesions were isointense to grey matter on the T1-weighted images, hypointense on the T2-weighted images, and showed strong uniform enhancement with contrast-enhanced imaging. Diffuse meningeal enhancement was observed in 6 patients but none had associated brain edema. In the contrast-enhanced CT images of 3 patients, the lesions appeared hyperdense relative to the contralateral mirror area. Additionally, the radiological contours of the lesions were similar to those observed in the MR images (Figure 4). No patient in this study had bony abnormalities. The imaging findings are summarized in Table 2.

**Table 2:** Route, location and radiological features of IOIS with retro-orbital involvement.

Patient No.	Side	Route of involvement	Location of retro-orbital lesions	MR T1	MRT2	MR Gd-DTPA	CECT density
1	left	SOF	MCF	iso	low	++	high
2	left	SOF, IOF	CS, MCF, PPF, ITF	iso	low	+++	NA
3	right	SOF	CS	iso	low	++	NA
4	left	SOF	MCF	iso	low	++	NA
5	left	SOF	CS, MCF	iso	low	+++	NA
6	left	SOF, IOF, OC	CS, MCF, PPF	iso	low	++	high
7	left	SOF, OC	CS, MCF	iso	low	++	high
8	right	SOF	CS, MCF	iso	low	+++	NA

Abbreviations: CECT, contrast-enhanced computed tomography; MR, magnetic resonance; Gd-DTPA, Gadopentetate dimeglumine; SOF, Superior orbital fissure; IOF, inferior orbital fissure; OC, optic canal; CS, Cavernous sinus; PPF, pterygopalatine fossa; ITF, infratemporal fossa; MCF, middle cranial fossa; ++, moderate enhancement; +++, strong enhancement; iso, isointense; low, low intensity; NA, not available.

### Pathological Findings

**Figure 5:**
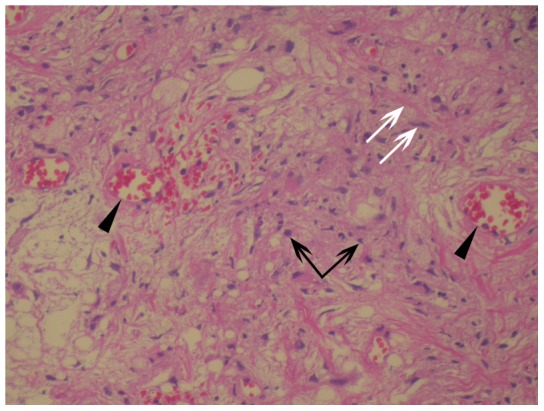
Histological findings of idiopathic orbital inflammation syndrome with retro-orbital involvement. This figure shows the results of haematoxylin and eosin staining. Note the presence of inflammatory cells (black arrows) that are associated with many thin-walled vessels (black arrowheads) and extensive fibrosis (white arrows). Magnification = 200×.

In all 8 patients with retro-orbital involvement, histopathological analysis revealed extensive fibrosis with new vessel growth. Fibrosis was associated with nonspecific infiltration of a few inflammatory cells including lymphocytes, histiocytes, neutrophils, plasma cells, and macrophages. Diffuse inflammation with marked sclerosis and hyalinization is consistent with the diagnosis of the sclerosing subtype of IOIS in all 8 patients (Figure 5).

### Treatment Outcomes

Of the 8 patients treated with systemic steroids in the study, 1 (12.5%) had complete symptom relief, 4 (50%) experienced partial relief, and 3 (37.5%) had no relief. During a mean follow-up time of 19 months, 6 patients (75%) had no recurrence or marked disease progression and 2 patients (25%) experienced increasing vision deterioration. There were no co-morbidities during or after systemic steroid therapy, with the exception of 2 patients who reported weight gain.

## Discussion

As the name suggests, IOIS is usually restricted to the orbit. Retro-orbital involvement, however, can occur. In our study, “retro-orbital” represents all regions behind the orbital apex. In a series of 90 consecutive patients with IOIS, Clifton *et al* reported 8 (8.8%) with CT evidence of intracranial involvement [3]. Our study, which based a diagnosis on radiological (MR and CT) findings, reports a similar frequency. The real frequency of retro-orbital involvement in IOIS is uncertain in our patients, as the false negative rate of imaging studies is unknown. Cranial nerve palsy is very common in patients with IOIS with retro-orbital involvement. However, cranial nerve palsy itself is not a reliable indicator of retro-orbital involvement because many patients with IOIS with orbital apex involvement but not retro-orbital involvement also have cranial nerve palsy. The cavernous sinus and the middle cranial fossa are the two most common locations of retro-orbital involvement. Inflammation can spread into these structures through three major openings: the superior orbital fissure, the inferior orbital fissure, and the optic canal. The superior orbital fissure is connected to the cavernous sinus and middle cranial fossa, the optic canal is located medial to the superior orbital fissure, and the inferior orbital fissure is connected to the orbit through the infratemporal and pterygopalatine fossa [4]. Of the three major openings, the superior orbital fissure is the most common route of involvement of the cavernous sinus and the middle cranial fossa [5]. Our findings are consistent with other reports in the literature [5]; the lesions of all 8 patients with retro-orbital involvement had extended through the superior orbital fissure into the cavernous sinus in 6 patients, and into the middle cranial fossa in 7 patients. Moreover, with the exception of 1 patient with perineuritis of the optic nerve, the other 7 patients had orbital apex lesions, suggesting that special attention needs to be given to retro-orbital structures when IOIS involves the orbital apex.

Radiological evaluation of IOIS includes both CT and MR imaging. CT is the preferred method because of the inherent contrast of the orbital fat, muscle, bony structures, and air in the adjacent paranasal sinuses [2]. Although some authors have reported that CT imaging is less sensitive than MRI [6], contrast-enhanced CT can detect differences in the soft tissue of the cavernous sinus and superior orbital fissure. Additionally, the radiological contours revealed using contrast-enhanced CT are similar to those obtained using MR imaging (as was observed in 3 of our patients who underwent both CT and MR imaging). Because the high signal intensity of orbital fat can easily mask subtle areas of inflammation or tissue with enhanced intensity, it is important to employ fat saturation when using contrast-enhanced imaging techniques [7]. Some authors have suggested that diffusion-weighted imaging (DWI) may help to differentiate between IOIS, orbital lymphoid lesions, and orbital cellulitis [8].

Neuroimaging is crucial for the diagnosis of retro-orbital involvement in cases of IOIS [9]. Retro-orbital lesions on T1-weighted images are isointense to grey matter, while on T2-weighted images they display lower signal intensity. Based on the histopathological findings of our study, the relative hypointensity of lesions on T2-weighted images may be due to the extensive fibrosis. In our patients, the lesions showed intense enhancement after administration of Gd-DTPA on MR imaging, which may be related to the histological findings of inflammatory cells and increased vascularity. Using contrast-enhanced CT, the retro-orbital lesions also appeared hyperdense relative to the contralateral mirror area.

The radiological features of Tolosa-Hunt syndrome (THS) are similar to those of IOIS with retro-orbital involvement [10]. THS is characterized by painful ophthalmoplegia, which is caused by inflammation of the cavernous sinus [11]. IOIS with retro-orbital involvement and THS seem to be part of a spectrum of disorders that have diverse locations but similar histological and imaging findings [10].

After periorbital pain (headache), cranial nerve palsies and decreased visual acuity are the next two most-frequent symptoms of IOIS with retro-orbital involvement, both of which may result from compression of the orbital apex and cavernous sinus by inflammatory infiltrate. In our study, the sclerosing subtype of IOIS had a poor therapeutic outcome, which is consistent with the other reports in the literature [12]. Administration of high-dose “pulsed” intravenous corticosteroids resulted in a substantial clinical improvement for most patients with ocular inflammation; this regimen may also be more effective than orally administered corticosteroids in patients with IOIS with retro-orbital involvement [13]. Because of incomplete collection of clinical data, our study did not address several important questions such as the incidence of deficits in color perception or the possible relationship between immunoglobulin G4 (IgG4)-positive plasma cells and the sclerosing subtype of IOIS.

In most patients, correlation of radiological features with the clinical presentation allows a diagnosis of IOIS with retro-orbital involvement and hence obviates the need for a biopsy. However, some radiological and clinical features of IOIS with retro-orbital involvement overlap with other diseases. CT and MR imaging, though very useful, do not always readily distinguish among the different entities. The differential diagnoses for the radiological findings include lymphoma, metastasis, meningioma, sarcoidosis, and infection [14], [15], [16].

## Conclusion

In summary, retro-orbital involvement in IOIS is more frequent when the disease is present in the orbital apex. Involvement of the retro-orbital area is an important sign that should not be overlooked by radiologists and ophthalmologists. The superior orbital fissure is the most common route through which inflammation spreads beyond the orbit. The two locations that become involved most frequently are the cavernous sinus and the middle cranial fossa.
